# Pathogenic Mechanisms in Centronuclear Myopathies

**DOI:** 10.3389/fnagi.2014.00339

**Published:** 2014-12-19

**Authors:** Heinz Jungbluth, Mathias Gautel

**Affiliations:** ^1^Neuromuscular Service, Department of Paediatric Neurology, Evelina Children’s Hospital, St Thomas’ Hospital, London, UK; ^2^Department of Basic and Clinical Neuroscience, Institute of Psychiatry, Psychology and Neuroscience (IoPPN), King’s College London, London, UK; ^3^Randall Division of Cell and Molecular Biophysics and Cardiovascular Division, King’s College London BHF Centre of Research Excellence, London, UK

**Keywords:** centronuclear myopathy, myotubular myopathy, *MTM1* myotubularin gene, *DNM2* dynamin-2 gene, *BIN1* bridging integrator-1/amphiphysin-2 gene, *RYR1* ryanodine receptor-1 gene, *TTN titin* gene, autophagy

## Abstract

Centronuclear myopathies (CNMs) are a genetically heterogeneous group of inherited neuromuscular disorders characterized by clinical features of a congenital myopathy and abundant central nuclei as the most prominent histopathological feature. The most common forms of congenital myopathies with central nuclei have been attributed to X-linked recessive mutations in the *MTM1* gene encoding myotubularin (“X-linked myotubular myopathy”), autosomal-dominant mutations in the *DNM2* gene encoding dynamin-2 and the *BIN1* gene encoding amphiphysin-2 (also named bridging integrator-1, BIN1, or SH3P9), and autosomal-recessive mutations in *BIN1*, the *RYR1* gene encoding the skeletal muscle ryanodine receptor, and the *TTN* gene encoding titin. Models to study and rescue the affected cellular pathways are now available in yeast, *C. elegans*, drosophila, zebrafish, mouse, and dog. Defects in membrane trafficking have emerged as a key pathogenic mechanisms, with aberrant T-tubule formation, abnormalities of triadic assembly, and disturbance of the excitation–contraction machinery the main downstream effects studied to date. Abnormal autophagy has recently been recognized as another important collateral of defective membrane trafficking in different genetic forms of CNM, suggesting an intriguing link to primary disorders of defective autophagy with overlapping histopathological features. The following review will provide an overview of clinical, histopathological, and genetic aspects of the CNMs in the context of the key pathogenic mechanism, outline unresolved questions, and indicate promising future lines of enquiry.

## Introduction

Centronuclear myopathies (CNMs) are a genetically heterogeneous group of inherited neuromuscular disorders characterized by clinical features of a congenital myopathy and abundant central nuclei as the most prominent histopathological feature [for review, see Jungbluth et al. (2008)]. CNMs are genetically widely heterogeneous and have been attributed to X-linked recessive mutations in *MTM1* encoding myotubularin [“X-linked myotubular myopathy (XLMTM)”] (Laporte et al., 1996), autosomal-dominant mutations in *DNM2* encoding dynamin-2 (Bitoun et al., 2005) and the *BIN1* gene encoding amphiphysin-2 (also named bridging integrator-1, BIN1, or SH3P9) (Bohm et al., 2014), and autosomal-recessive mutations in *BIN1* (Nicot et al., 2007), *RYR1* encoding the skeletal muscle ryanodine receptor (Wilmshurst et al., 2010), and *TTN* encoding titin (Ceyhan-Birsoy et al., 2013).

Whilst histopathological abnormalities other than abundant central nuclei are not typically observed in association with *MTM1* and *BIN1* mutations, the common occurrence of central nuclei, marked variability in fiber size and cores with some of the other genetic backgrounds, in particular recessive *RYR1* (Bevilacqua et al., 2011) and *TTN* mutations (Ceyhan-Birsoy et al., 2013; Chauveau et al., 2014a), have challenged the concept of CNM as a “pure” entity (Romero, 2010) and have suggested a continuum with other congenital myopathies, in particular the core myopathies [for review, see Jungbluth et al. (2011)] and congenital fiber type disproportion (CFTD) (Clarke et al., 2010). The neuromuscular disorder due to dominant mutations in the *CCDC78* gene encoding Coiled-Coil Domain-Containing 78 (Majczenko et al., 2012) is another example of a congenital myopathy difficult to classify on histopathological grounds due to the common occurrence of internalized nuclei and cores, containing sarcoplasmic aggregated CCDC78, desmin (DES), actin (ACTA1), and RYR1.

Models to study and rescue the cellular pathways affected in various forms of CNM are now available in yeast (Parrish et al., 2004; Cebollero et al., 2012), *C. elegans* (Dang et al., 2004; Zou et al., 2009; Neukomm et al., 2011), drosophila (Velichkova et al., 2010), zebrafish (Dowling et al., 2009; Gibbs et al., 2013), mouse (Buj-Bello et al., 2002; Durieux et al., 2010b; Pierson et al., 2012; Fetalvero et al., 2013; Reifler et al., 2014), and dog (Beggs et al., 2010; Bohm et al., 2013). Based on observations in these models, several pathogenic mechanisms have now been suggested, including abnormalities of triads and calcium handling (Al-Qusairi et al., 2009; Dowling et al., 2009; Bohm et al., 2014), as well as defects of the neuromuscular junction (Robb et al., 2011; Dowling et al., 2012), satellite cells (Lawlor et al., 2012), mitochondria, and the DES cytoskeleton (Hnia et al., 2011).

Alterations of the autophagy pathway have recently emerged as a pathogenic mechanism common to different genetic forms of CNM (Al-Qusairi et al., 2013; Fetalvero et al., 2013). Autophagy is a fundamental cellular degradation pathway conserved throughout evolution with important roles in the removal of defective proteins and organelles, defense against infections and adaptation to changing metabolic demands (Mizushima, 2007; Sandri et al., 2013; Wang and Robbins, 2013). Autophagy is physiologically enhanced in neurons and muscle, and in conjunction with the ubiquitin–proteasome pathway, plays a major role in the pathogenesis of muscle atrophy (Sandri, 2013). The autophagy pathway involves several tightly regulated steps, evolving from the initial formation of phagophores to autophagosomes, whose fusion with lysosomes results in the final structures of degradation, autolysosomes [for review, see Mizushima (2007)]. The recent implication of defective autophagy in CNM corresponds to the recognition of its increasing role in a wide range of neuromuscular disorders with both primary and secondary autophagy defects (Merlini and Nishino, 2014). The observation of histopathological features closely resembling CNM in Vici syndrome (McClelland et al., 2010), a severe human multisystem disorders due to recessive mutations affecting the key autophagy regulator epg5 (Cullup et al., 2013), provides additional support for a link between the CNMs and the autophagy pathway.

The majority of the defective proteins implicated in the CNMs to date – myotubularin, dynamin-2, and amphiphysin-2 – are involved in various aspects of membrane trafficking and remodeling relevant to essential cellular processes including endocytosis, intracellular vesicle trafficking, and autophagy [for review, see Cowling et al. (2012)], suggesting a pathogenic “master mechanism” upstream of the more specific downstream pathogenic mechanisms outlined above. However, a link between membrane trafficking and other genes implicated in the CNMs is not immediately obvious, and the communality of clinico-pathological features between *MTM1*, *DNM2*, and *BIN1*-related CNM on one hand and the more recently reported forms due to recessive mutations in *RYR1* and *TTN* on the other hand remains currently unaccounted for on the molecular level.

**Figure 1:**
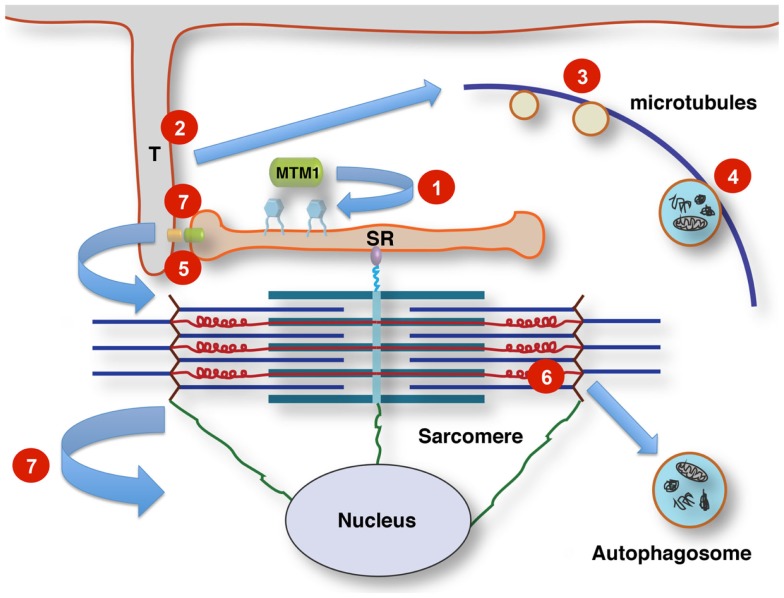
**Tentative links between membrane signaling, sarcomere activity, and nuclear positioning**. T-tubules (T) link the sarcolemma to the sarcoplasmic reticulum (SR) at the triads by contacts between dihydropyridine receptors (small orange oval) and ryanodine receptors (small green oval). Phosphoinositol phosphates (PIP; blue hexagonal symbols at SR membrane) are turned over by the lipid phosphatase myotubularin, regulating membrane dynamics, and PIP-dependent downstream signaling. This affects multiple pathways, including autophagy. Altered membrane remodeling and microtubular transport will converge on these pathways. Defects affecting Titin’s scaffolding role concerning multiple components of the protein quality control machinery impinge on contractile function, sarcomere turnover and possibly sarcomere-nuclear links. Nuclei and sarcomeres are joined by peripheral cytoskeletal networks, including desmin intermediate filaments and nesprin via “transmembrane actin-associated nuclear links” (Luxton et al., 2011). **1**, Mutations affecting lipid phosphatase activity of myotubularin (MTM1 and MTMR14); **2**, mutations in components of the membrane remodeling machinery (BIN1); **3**, defects in vesicular traffic or microtubule dynamics (DNM2); **4**, mutations in the endosomal–autophagosomal–lysosomal pathway (EPG5); **5**, defective calcium homeostasis and excitation–contraction coupling (RYR1); **6**, defective sarcomeric maintenance and protein quality control (TTN); **7**, disrupted nuclear cytoskeleton links, abnormal nuclear positioning and possibly impaired triad function via impaired microtubule/centriole function (CCDC78).

The following review will give an overview of the key clinical, histopathological, and genetic aspects of the different forms of CNM, outline pathogenic mechanisms where already known, with a particular emphasis on defects in membrane trafficking and autophagy, and summarize unresolved questions and future lines of enquiry. Table 1 summarizes the genes and proteins implicated in the CNMs and outlines their main function(s) where known. Figure 1 illustrates tentative links between the different pathways implicated in the CNMs.

**Table 1:** **Genes and proteins implicated in various forms of centronuclear myopathy (CNM)**.

Gene	Inheritance	Protein	Principal function(s)	Main pathogenic effects in muscle
*MTM1*	XL	Myotubularin	PI3P regulation Membrane formation/trafficking Endocytosis Endo(lyso)some formation	Abnormal nuclear positioning Abnormalities of triad positioning and function Abnormal autophagy Abnormal cytoskeletal architecture Abnormal mitochondrial positioning Autophagosome formation
*DNM2*	AD	Dynamin-2	Membrane formation/trafficking Vesicle formation and fission	Abnormal nuclear positioning Abnormalities of triad positioning and function Abnormal autophagy Abnormal cytoskeletal architecture Abnormal mitochondrial positioning
*BIN1*	AR, AD	Amphiphysin-2	Membrane remodeling	Abnormalities of nuclear positioning, triad assembly and function
*RYR1*	AR	Skeletal muscle ryanodine receptor	Sarcoplasmic reticulum calcium release	Abnormal nuclear positioning Abnormalities of triad assembly and function Abnormal SR calcium release
*TTN*	AR	Titin	Elastic link between actin and myosin filaments Organizer of Z-disk and M-band assembly Organizer of myosin filament, possibly by regulating myosin motor domains Mechanosensor Signaling scaffold organizing ubiquitin–proteasome and autophagy–lysosomal protein turnover	Abnormal sarcomere assembly and turnover Disrupted force transmission Abnormal myosin force generation Abnormal transcriptional regulation
*MTMR14*	AR? Digenic?	hJUMPY	PI3P regulation Membrane formation/trafficking	Abnormal nuclear positioning Abnormal excitation–contraction coupling Abnormal autophagy
*CCDC78*	AD	Coiled-coil domain- containing protein 78	Centriole biogenesis?	Abnormal nuclear positioning Core formation?

## *MTM1*-Related CNM (“X-Linked Myotubular Myopathy”)

Centronuclear myopathy due to X-linked recessive mutations in the myotubularin *(MTM1)* gene (also commonly referred to as XLMTM) is a rare congenital myopathy that affects approximately 2/100000 male births per year [for review, see Jungbluth et al. (2008)].

X-linked myotubular myopathy is characterized by a severe phenotype in males with often antenatal onset, profound hypotonia, and weakness at birth with associated severe respiratory and bulbar involvement necessitating invasive ventilation and nasogastric tube feeds. Extraocular muscle involvement is common. The condition is usually fatal within the first year of life but a proportion of more mildly affected males may survive into adolescence or adulthood, sometimes even without requiring constant ventilatory support. Although profound muscle involvement is the most dramatic and earliest feature of myotubularin deficiency, long-term survivors show additional organ manifestations such as hepatic peliosis suggestive of a multisystem disorder (Herman et al., 1999), indicating that myotubularin does play a vital role in tissues other than muscle. Moreover, despite muscle atrophy and weakness, males with XLMTM typically exhibit signs of macrosomia consistent with an overgrowth syndrome (Leguennec et al., 1988; Joseph et al., 1995), suggesting a differential effect of myotubularin deficiency on muscle and other growth pathways. A dilated cardiomyopathy has been reported in two adult brothers with a mild form of XLMTM (Yu et al., 2003), raising the possibility of a cardiac phenotype in long-term survivors that remains to be systematically evaluated. Histopathological features in addition to numerous central nuclei include type 1 predominance and hypotrophy, a region devoid of myofibrils surrounding the central nucleus and necklace fibers (Romero and Bitoun, 2011), but additional sarcomeric disorganization or overt cores are unusual in contrast to the *DNM2*-, *RYR1*- and *TTN*-related forms.

More than 300 *MTM1* mutations have been identified to date (Laporte et al., 2000; Herman et al., 2002; Biancalana et al., 2003; Tsai et al., 2005), distributed throughout the entire coding sequence and with only few recurrent substitutions. Genotype–phenotype studies have been limited due to the private nature of many *MTM1* mutations, however, not unexpectedly truncating mutations usually give rise to the more severe phenotype whilst non-truncating mutations outside the myotubularin catalytic domain have been associated with milder presentations. Markedly skewed X-inactivation in manifesting females (Jungbluth et al., 2003), as well as complex rearrangements involving the *MTM1* locus have also been recently reported (Trump et al., 2011; Amburgey et al., 2013).

Myotubularin defines a family of 14 phosphoinositide phosphatases in mammals [for review, see Laporte et al. (1998, 2001, 2003), Begley and Dixon (2005), Clague and Lorenzo (2005), Robinson and Dixon (2006), and Amoasii et al. (2012)], two of which, MTMR2 and MTMR13, have also been implicated in different forms of Charcot-Marie-Tooth (CMT) disease, CMT4B1 (Berger et al., 2002) and CMT4B2 (Azzedine et al., 2003), respectively. In addition to the catalytic and enzymatically active domain, myotubularin contains four other domains, including a coiled–coiled domain involved in homo- and heterodimer formation. Apart from the recognized interaction with DES, only little is known about interactions with other proteins in skeletal muscle.

Myotubularin dephosphorylates phosphatidylinositol 3- phosphate [PI(3)P] and phosphatidylinositol 3,5-phosphate [PI(3,5)P] [(Blondeau et al., 2000; Taylor et al., 2000; Tronchere et al., 2004); for review, see Tronchere et al. (2003), Robinson and Dixon (2006), and Rohde et al. (2009)], second messengers with a crucial role in membrane trafficking whose production is under the control of specific phosphatidylinositide kinases. In skeletal muscle, the main generator of PI(3)P is the PI3 kinase PIK3C3 (Backer, 2008; Meijer and Klionsky, 2011), a key regulator of a wide range of cellular processes including autophagy, in particular formation and maturation of autophagosomes (Funderburk et al., 2010).

The fundamental role of myotubularin and its orthologs in PI3P regulation, endocytosis, and endo(lyso)somal function has been documented in drosophila, *C. elegans*, zebrafish, mouse, and higher mammalian models of myotubularin deficiency [for review, see Cowling et al. (2012)]. Zebrafish morphants following mtm1 morpholino knockdown show abnormal motor behavior and reproduce some of the histopathological features also seen in human XLMTM, associated with increased PI3P levels in muscle (Dowling et al., 2009). In contrast to human XLMTM, the mtm1 knockout mouse develops muscle weakness and atrophy only in the postnatal period, suggesting an effect of myotubularin deficiency on muscle maintenance rather than muscle development (Buj-Bello et al., 2002). Secondary abnormalities of T-tubules, sarcoplasmic reticulum (SR) and the triads (Al-Qusairi et al., 2009; Dowling et al., 2009; Beggs et al., 2010; Toussaint et al., 2011) and, less frequently, abnormalities of intermediate filaments and mitochondrial dynamics (Hnia et al., 2011) have been reported as an important common downstream effect of myotubularin deficiency in zebrafish, mouse, dog, and humans.

Initiation of autophagy, in particular formation of autophagosomes and autophagosome–lysosome fusion, depends on PI3P synthesis (Vergne and Deretic, 2010; Cebollero et al., 2012), and the concerted interaction of autophagy-related (Atg) proteins at the phagophore assembly site (PAS) (Lamb et al., 2013; Ge et al., 2014); considering the important role of myotubularin in regulating PI3P levels in muscle, it is not surprising that alterations of muscle autophagy have now been reported in animal models of XLMTM: In particular, a marked disturbance of autophagy has been reported in zebrafish following double knockdown of the myotubularin family members *MTM1* and *MTMR14* (Dowling et al., 2010), the latter also known as Jumpy and implicated in very rare digenic forms of CNM (Tosch et al., 2006). Mytotubularin deficiency has also been associated with increased mTORC1 activity, disconnection between starvation and autophagy induction (Fetalvero et al., 2013), increased IGF1R/Akt signaling, upregulation of atrogenes and an increase in autophagy markers in the mtm1 knockout mouse (Al-Qusairi et al., 2013), indicating both up- and downstream effects of murine myotubularin deficiency on the autophagy pathway that are potentially amenable to mTOR inhibition with Rapamycin (Fetalvero et al., 2013) and adeno-associated virus (AAV)-mediated delivery of functional myotubularin (Al-Qusairi et al., 2013). Interestingly, a recent study reporting a muscle-specific conditional knockout of PIK3C3, the phosphatidylinositide 3-kinase critical for PI3P levels in muscle, indicates marked autophagolysosomal abnormalities with histopathological features more suggestive of a muscular dystrophy rather than CNM (Reifler et al., 2014). These observations suggest the autophagy pathway and its upstream regulators as potential therapeutic targets in CNM and, possibly, other forms of neuromuscular disorders.

## *DNM2*-Related CNM

Dominantly inherited *DNM2*-related CNM is usually much milder than X-linked and recessive forms of CNM although more severe presentations have been reported [for review, see Jungbluth et al. (2008)]. Onset is typically in adolescence or early adulthood, featuring predominant proximal weakness with additional distal involvement particularly in the lower limbs, ptosis with external ophthalmoplegia, and a stable or slowly progressive course. Exertional myalgia may be the presenting feature before the evolution of overt weakness and muscle hypertrophy, occasionally localized, has been observed (Liewluck et al., 2010). Specific dominant intermediate (CMTDIB) and axonal forms of CMT disease (CMT2), respectively, are allelic conditions (Zuchner et al., 2005; Fabrizi et al., 2007). In addition to myopathic changes, EMG and nerve conduction studies may show mild signs of axonal peripheral nerve involvement also in *DNM2*-related CNM patients (Fischer et al., 2006; Echaniz-Laguna et al., 2007), suggesting a clinical continuum between myopathic and neuropathic manifestations of *DNM2* mutations. Other *DNM2*-mutated patients may feature additional neutropenia (Liewluck et al., 2010) or cataracts (Jungbluth et al., 2010), suggesting a role of dynamin-2 in tissues other than muscle, as well as clinical overlap with multisystem disorders due to primary autophagy defects such as *EPG5*-related Vici syndrome (Cullup et al., 2013), where cataracts and hematological abnormalities are common. Homozygosity for the *DNM2* Phe379Val missense mutation has been recently associated with a congenital lethal syndrome in humans (Koutsopoulos et al., 2013). Histopathological features in addition to centralized nuclei may include type 1 predominance, typical sarcoplasmic radial strands surrounding the central nuclei, increases in connective tissues and cores (Fischer et al., 2006; Schessl et al., 2007; Jeub et al., 2008; Hanisch et al., 2011; Bohm et al., 2012a; Catteruccia et al., 2013).

The *DNM2* gene is one of three members of the dynamin family (Praefcke and McMahon, 2004) and ubiquitously expressed, in contrast to *DNM1* that is mainly expressed in the brain, and *DNM3* expressed in brain and testes. *DNM2* encodes a large GTPase protein organized in five functional domains, an N-terminal GTPase domain, a middle domain (MD), a pleckstrin homology (PH) domain, a GTPase effector domain, and a C-terminal proline rich domain (PRD) (McNiven, 2005). Through its PH and PRD domains, dynamin-2 binds to phosphoinositides and SH3 domain proteins such as amphiphysin, respectively. Dominant mutations affecting the dynamin-2 MD have been associated with a mild phenotype of CNM (Bitoun et al., 2005), whilst more severe presentations with neonatal onset have been attributed to heterozygous *de novo* dominant mutations affecting the PH domain (Bitoun et al., 2007; Jungbluth et al., 2010). The dynamin-2 PH domain is also predominantly affected by *DNM2* mutations causing primary neuropathic phenotypes. A recurrent *DNM2* mutation (c.1393C > T; p.Arg465Trp) has been identified in a number of unrelated autosomal-dominant pedigrees with a mild form of CNM.

Dynamins are involved in membrane fission, and the role of various isoforms including dynamin-2 in clathrin-dependent and independent endocytosis, vesicle formation and processing (Jones et al., 1998; Praefcke and McMahon, 2004; Durieux et al., 2010a) has been documented in various models of dynamin deficiency. Additional roles have been proposed in the microtubule network, actin cytoskeleton assembly (Gu et al., 2010), and centrosome cohesion (Thompson et al., 2004), the latter of potential relevance for the nuclear abnormalities observed in *DNM2*-related CNM.

Murine models of the common human *DNM2* R465W dominant CNM mutation do replicate aspects of the human phenotype, and not unexpected considering the close links between endocytic and autophagic pathways, show variable abnormalities of autophagy: Durieux et al. (2010b, 2012) demonstrated a slowly progressive myopathy with upregulation of genes involved in ubiquitin–proteosome (UPS) and autophagy pathways in a heterozygous knock-in mouse model of the common CMT-associated heterozygous *DNM2* mutation R465W. Mice homozygous for the R465W mutation showed a severe phenotype similar to what has been observed in other mouse models of dysregulated autophagy (Durieux et al., 2012), characterized by increased glycogen storage, hepatomegaly, hypoglycemia, and early lethality. The same mice showed microscopic evidence of delayed autophagosome maturation and of reduced autophagic flux on *in vitro* studies. Another mouse model generated by intramuscular AAV injection of mutant R465W–DNM2 generated histopathological abnormalities and T-tubule defects similar to those observed in humans and animal models of other forms of CNM, suggesting a muscle maintenance defect as the principal abnormality also in *DNM2*-related CNM. An intriguing and potentially therapeutically exploitable link between *DNM2*- and *MTM1*-related pathways has been recently indicated by demonstrating rescue of the XLMTM phenotype through dynamin-2 reduction in mice (Cowling et al., 2014).

## *BIN1*-Related CNM

Autosomal-recessive *BIN1*-related CNM has only been reported in a small number of families associated with a mild to moderate phenotype characterized by early-childhood onset, extraocular muscle involvement and slowly progressive muscle weakness, and atrophy (Nicot et al., 2007; Claeys et al., 2010). However, more severe, early-onset lethal (Nicot et al., 2007) and rapidly progressive presentations due to homozygous *BIN1* mutations affecting splicing have been reported (Bohm et al., 2010, 2013). Dominant inheritance of *BIN1* mutations has also been recently recognized (Bohm et al., 2014). In addition to central nuclei, type 1 fiber-type predominance may be an additional feature, but sarcomeric disorganization and core-like areas are uncommon [for review, see Jungbluth et al. (2008)].

*BIN1* encodes amphiphysin-2, a protein belonging to the BAR (Bin/Amphiphysin/Rvs) domain-containing family of proteins (Peter et al., 2004) involved in various key cellular processes including membrane recycling and endocyotosis [for review, see Prokic et al. (2014)]. Corresponding to other proteins implicated in the CNMs, BIN1 also contains a phosphoinositide-binding domain and is involved in T-tubule formation. Mutations affecting the *BIN1* BAR domain impair membrane tubulation and result in structural abnormalities (Wu et al., 2014). *BIN1* is ubiquitously expressed but subject to tissue-specific alternative splicing, whereas amphiphysin 1, the other member of the amphiphysin family, is mainly expressed in brain. *BIN1* downregulation has been associated with cancer progression and cardiac disease, whereas *BIN1* overexpression has been linked to an increased risk for late-onset Alzheimer disease [for review, see Prokic et al. (2014)].

The essential role of amphiphysins and their orthologs in endocytosis, membrane remodeling and recycling has been documented in drosophila and *C. elegans* models of amphiphysin deficiency [for review, see Cowling et al. (2012)]. A recent Bin1-deficient zebrafish model of *BIN1*-related CNM reproduces the histopathological features of the human phenotype, and indicates abnormal calcium release resulting from aberrant triad formation as an important pathogenic mechanism downstream of the principal membrane remodeling abnormality (Smith et al., 2014). The T-tubule and triadic abnormalities observed in the Bin1-deficient zebrafish model are similar to those observed in *MTM1*- and *DNM2*-related CNM (Toussaint et al., 2011), indicating a shared pathogenic mechanism due to implication of the defective proteins in the same pathway. *BIN1*-deficient mice show early lethality (Muller et al., 2003), but murine skeletal muscle has not yet been thoroughly analyzed. A recent mouse model of Bin1 depletion in the heart shows abnormalities of T-tubule folding resulting in free diffusion of local extracellular calcium and potassium ions, prolonged action-potential duration and increased susceptibility to ventricular arrhythmias (Hong et al., 2014).

BIN1 deficiency has not yet been associated with defects in the autophagy pathway, however, it is of note that structurally related BAR domain-containing proteins such as SH3P2 translocate to the PAS following autophagy induction and appear to play a role in autophagosome formation (Zhuang et al., 2013).

## *RYR1*-Related CNM

Recessive mutations in *RYR1* are another cause of congenital myopathies with central nuclei (Wilmshurst et al., 2010). *RYR1* mutations are one of the most common causes of inherited neuromuscular disorders, ranging from the malignant hyperthermia susceptibility (MHS) trait without any associated weakness to various congenital myopathies, including mainly dominantly inherited Central Core Disease (CCD) as well as mainly recessively inherited Multi-minicore Disease (MmD) [for review, see Jungbluth et al. (2011)], CFTD (Clarke et al., 2010), and CNM (Wilmshurst et al., 2010). The genetics of *RYR1*-related myopathies are not infrequently complex, occasionally with two clearly pathogenic *RYR1* mutations occurring on the same allele or running independently in the same family, possibly accounting for the wide phenotypical variability and variable penetrance (Klein et al., 2012). There is substantial clinical and pathological overlap between MmD, CFTD, and CNM due to recessive *RYR1* mutations, and it appears appropriate to view these conditions as part of a recessive *RYR1-*related continuum rather than completely distinct entities.

Clinically, *RYR1*-related CNM is of intermediate severity compared to other genetic forms, with facial weakness, external ophthalmoplegia, predominantly proximal muscle involvement but less pronounced bulbar or respiratory impairment (Wilmshurst et al., 2010). There is however, a more severe end of the spectrum, with some profoundly affected males showing clinical presentations indistinguishable from the XLMTM phenotype. Patients with *RYR1*-related CNM show a marked tendency to improve over time, even following an initially severe presentation, a feature also in other recessive *RYR1*-related myopathies (Bohm et al., 2012b) that remains currently unexplained.

On the pathological level, central and multiple internalized nuclei are often the principal histopathological feature when muscle biopsy is performed early in life (Jungbluth et al., 2007), but other histopathological features typically associated with recessive *RYR1*-related myopathies – marked type 1 predominance or uniformity, fiber type disproportion and cores – may evolve over time (Bevilacqua et al., 2011).

In contrast to dominantly inherited MHS and CCD where most features can be explained by abnormal calcium release from the mutant RyR1 channel [for review, see Treves et al. (2005)], the pathogenesis of recessive *RYR1*-related myopathies is currently only partly understood and probably more complex. Recessive *RYR1* genotypes, often featuring compound heterozygosity for *RYR1* missense and truncating mutations, result in reduced expression of the RyR1 protein and secondary reduction of the DHPR receptor, its principal ligand (Wilmshurst et al., 2010; Bevilacqua et al., 2011; Zhou et al., 2013). Additional upregulation of the IP3R receptor may be found in some cases, an observation currently of uncertain significance (Zhou et al., 2013). Whilst the concomitant reduction of RyR1 and DHPR and the resulting excitation–contraction (EC) coupling defect are likely to explain the weakness, muscle atrophy as well as histopathological features of fiber-type disproportion and centralized nuclei are not readily explained by alterations of calcium release in recessive *RYR1*-related myopathies.

In contrast to other genetic forms of CNM, defects in membrane trafficking and autophagy have not been implicated in recessive *RYR1*-related CNM. However, it is of note that marked autophagy abnormalities have been observed in mice following induced reduction of the DHPR receptor (Pietri-Rouxel et al., 2010), a secondary feature also in recessive *RYR1*-related myopathies. In addition, the recent implication of annexin-1 and annexin-5, members of the annexin family of proteins that bind to phospholipid membranes in a calcium-dependent manner, in autophagosome maturation (Ghislat and Knecht, 2012) suggests a potential link between disturbed calcium homeostasis and autophagy regulation that may warrant further investigation. Effect of altered calcium release on autophagic pathways have been previously considered but with conflicting conclusions (East and Campanella, 2013).

## *TTN*-Related CNM

Recessive mutations in *TTN* encoding the giant sarcomeric ruler protein titin have recently been identified by next generation sequencing in five individuals selected from a cohort of 29 unrelated and genetically unresolved patients with a clinico-pathological diagnosis of CNM (Ceyhan-Birsoy et al., 2013). *TTN* mutations have recently also been indicated as one of the most common identifiable genetic causes of dilated cardiomyopathy (Herman et al., 2012), and have been implicated in a wide range of neuromuscular disorders, including late-onset tibial muscular dystrophy, limb girdle muscular dystrophy type 2J (LGMD2J), hereditary myopathy with early respiratory failure (HMERF), and early-onset myopathy with fatal cardiomyopathy [reviewed recently in Chauveau et al. (2014b)]. Although the functional relevance and clear genotype–phenotype correlations have been established for many unequivocally pathogenic *TTN* mutations, it is also important to bear in mind that truncating *TTN* variants are exceedingly common [over 6000 in the December 2014 release of the 1000 genomes database, and ca. 3% of controls reported in Herman et al. (2012)] and that pathogenicity is not always immediately evident even if those variants are truncating. This might be due to tissue-specific and developmentally regulated exon usage, especially in the extensively differentially spliced I-band region of titin (Bang et al., 2001), making some truncating variants penetrant in only a small subset of titin isoforms. But also, truncating mutations near the C-terminus appear, on the whole, to be recessive without an adult phenotype (Carmignac et al., 2007; Ceyhan-Birsoy et al., 2013; Chauveau et al., 2014a). The reasons for the recessive inheritance of truncating variants in constitutively expressed exons remains to be understood.

Clinical features of the five patients with *TTN*-related CNM in the study by Ceyhan-Birsoy et al. (2013) were characterized by early-childhood onset, generalized weakness, and respiratory impairment, but without evidence of cardiac involvement at the time of the last follow-up in childhood or late adolescence (5–19 years). In contrast to other genetic forms of CNM, extraocular muscles were spared and in one case CK levels were increased >1000 IU/l. Histopathological features included increases in connective tissue, fiber type disproportion, and type 1 predominance and hypotrophy. In contrast to *MTM1*-related CNM, but corresponding to findings in the *RYR1*-related form, central and internalized nuclei were typically multiple rather than single. Similar observations were made in the seminal paper on the recessive truncating *TTN*-linked early-onset Salih myopathy (Carmignac et al., 2007) and in four families with compound heterozygous *TTN* variants in Autosomal-Recessive Multi-minicore Disease with Heart Disease (AR MmD-HD) (Chauveau et al., 2014a). Patients with AR MmD-HD show marked centronucleation with additional morphological changes, notably the formation of protein aggregates and Z-disk streaming that show ultrastructural similarities to those found in myofibrillar myopathy. Patients also display various cardiac phenotypes, from left-ventricular non-compaction to septal defects (ASD and VSD) and dilated cardiomyopathy, in some instances requiring transplantation (Chauveau et al., 2014a). Additional findings of core-like areas on oxidative stain and myofibrillar disruption on EM, in particular Z-disk streaming and sarcomere disruption, suggest that *TTN*-related CNM and AR MmD-HD may be part of a *TTN*-related histopathological spectrum rather than a pure entity, again corresponding to observations in the *RYR1*-related form.

The pathogenesis of *TTN-*related CNM and in particular its association with pathways affected in other forms of CNM, if any, remains currently uncertain. Most mutations identified in *TTN*-related CNM give rise to significant C-terminal truncations, with or without the expression of disruptive missense variants, resulting in secondary reduction of interacting proteins such as nebulin and calpain-3 that may contribute to the phenotype. Calpain-3 is required for the normal recruitment of RyR1 receptors to the triad, a function that, if disturbed, may give rise to similar abnormalities of triad assembly and EC coupling as seen in other genetic forms of CNM. A common feature of *TTN*-linked AR MmD-HD and CNM is, however, the disruption of titin M-band linked interactions; of these, three are possibly mechanistically related to pathways linked to the “classical” CNM variants. Firstly, M-band titin links the sarcomere to the SR via its interactions with the giant protein obscurin (Bagnato et al., 2003; Kontrogianni-Konstantopoulos et al., 2003; Fukuzawa et al., 2008) and thus contributes to the organized integration of the EC-coupling machinery of T-tubules, junctional SR, and sarcomeres. Intriguingly, obscurin knockout mice also develop a myopathic phenotype with centralized nuclei and disordered SR (Lange et al., 2009). Secondly, the M-band associated kinase domain of titin is linked to the control of protein turnover via the autophagy cargo adaptors Nbr1 and SQSTM1 [reviewed in Gautel (2011)]. Lastly, mutations in the C-terminus of titin are linked to secondary calpain-3 deficiency also in the case of adult titinopathies (Udd, 2012), likely due to the abrogation of a calpain-3 binding site near the C-terminus of titin (Charton et al., 2010). While the connections between titin mutations, protein turnover, and abnormal nuclear positioning in titin-associated CNM-like myopathies are currently unclear, accumulating evidence suggests that protein turnover via autophagy and calpain-mediated turnover converge on M-band titin and that these connections are concerted with physical links to the SR and triad systems. If such links exist, it seems plausible that the ablation or functional disruption of titin-linked autophagy functions in M-band titinopathies (Chauveau et al., 2014a) result in partial phenotypic overlap with membrane-associated components of the autophagy machinery.

## Rare Congenital Myopathies with Central Nuclei

Congenital myopathies with features of CNM with or without additional histopathological abnormalities due to uncommon genetic backgrounds have been observed in isolated families.

Tosch and colleagues reported single heterozygous missense variants in hJUMPY (also known as MTMR14, a member of the myotubularin family) in two sporadic cases with features of CNM and uncertain inheritance (Tosch et al., 2006). Although both variants were demonstrated to reduce the enzymatic activity of hJUMPY, identification of an additional *DNM2* mutation in one patient suggests that a second mutation may be required for full manifestation of clinical features; this is also in keeping with the observation of a more severe phenotype in the MTM1–MTMR14 zebrafish double knockout compared to knockout of each single gene (Dowling et al., 2010).

Autosomal-dominant mutations in CCDC78 have also recently been identified in a single family characterized by core-like areas and increased internalized nuclei (Majczenko et al., 2012); CCDC78 encodes a skeletal muscle protein enriched in the perinuclear region and at the sarcolemma and possibly triad (Majczenko et al., 2012), suggesting a possible link with a pathogenic mechanism, abnormal triad assembly, and resulting disturbance of EC coupling, common to other forms of CNM. CCDC78 plays a key role in centriole biogenesis (Klos Dehring et al., 2013); the impaired function in CNM4 and the link to potential triad malfunction or abnormal nuclear positioning via impaired microtubule function is currently elusive.

## Conclusion and Outlook

Recent years have seen substantial advances in our understanding of the CNMs, in particular those due to mutations in *MTM1*, *DNM2*, and *BIN1*, encoding proteins intricately linked in various aspects of phosphoinositide metabolism and membrane trafficking, with aberrant T-tubule formation, abnormalities of triad assembly and disturbance of the EC machinery as the most important downstream effects studied to date. Abnormal autophagy has recently been recognized as another important pathogenic mechanism in different genetic forms of CNM, suggesting an intriguing link to primary disorders of defective autophagy with overlapping histopathological features. These findings have illustrated the role of defective pathways common to several genetic forms of CNM that may be potentially amenable to therapeutic intervention. It remains currently uncertain if the proteins encoded by genes more recently implicated in the CNMs, in particular *RYR1* and *TTN*, are involved with the same pathways or linked with altogether different mechanisms. The functional links between the genetic mechanisms implicated in CNM are tentative at the moment, and it has to be seen whether all myopathies clinically classified as CNM indeed join into a common pathomechanistic pathway. Although the mechanisms outlined above may at least partially explain the muscle weakness and atrophy observed in different forms of CNM, other aspects such as the consistent abnormality of nuclear positioning remain currently unaccounted for. The molecular machinery involved in nuclear positioning is currently only partially understood [for review, see Osorio and Gomes (2014)], but emerging evidence suggests that normal positioning of the nucleus is a prerequisite for its normal functioning (Metzger et al., 2012). Recent work has already suggested a link between N-WASP and BIN1-related nuclear positioning and triad organization (Falcone et al., 2014). Further investigation of the CNMs as a paradigm of disorders with nuclear positioning as the most prominent pathological hallmark will advance our understanding of the intricate interaction between the nucleus, microtubules and the actomyosin cytoskeleton (Luxton et al., 2011; Cadot et al., 2012), and delineate the importance of the interplay of these structures for cellular function in health and disease.

## Conflict of Interest Statement

The authors declare that the research was conducted in the absence of any commercial or financial relationships that could be construed as a potential conflict of interest.
